# Ethanol pharmacokinetics before and after sleeve gastrectomy and Roux-en-Y gastric bypass: a 3 year prospective study (the BAR-TRIAL)

**DOI:** 10.1038/s41366-026-02113-3

**Published:** 2026-06-17

**Authors:** Magnus Strømmen, Ola Dale, Christian Klöckner, Ida Tylleskär

**Affiliations:** 1 Centre for Obesity Research, Clinic of Surgery, St. Olavs Hospital, Trondheim University Hospital, Trondheim, Norway; 2 Department of Clinical and Molecular Medicine, Norwegian University of Science and Technology (NTNU), Trondheim, Norway; 3 Department of Circulation and Medical Imaging, Norwegian University of Science and Technology (NTNU), Trondheim, Norway; 4 Department of Psychology, Norwegian University of Science and Technology (NTNU), Trondheim, Norway; 5 Orkdal Department of Internal Medicine, St. Olavs Hospital, Trondheim University Hospital, Trondheim, Norway

**Keywords:** Metabolism, Obesity

## Abstract

**Objectives:**

To compare ethanol pharmacokinetics before and after Roux-en-Y gastric bypass (RYGB) and sleeve gastrectomy (SG) over a three-year period.

**Methods:**

This was a prospective, longitudinal, non-randomized controlled study at St. Olavs Hospital, Trondheim, including 33 adults without histories of alcohol use disorder (RYGB: *n* = 14; SG: *n* = 19). Participants underwent oral and intravenous ethanol challenge tests preoperatively and at 3, 12, and 36 months postoperatively. Primary outcomes included maximum plasma concentration (C_max_), time to reach maximum concentration (T_max_), and area under the curve to the last measurable concentration (AUC_last_).

**Results:**

The ethanol uptake after both surgical procedures was bioequivalent for AUC_last_, but not for C_max_ and T_max_. At 12 months, RYGB gave a 27% higher C_max_ and 31% shorter T_max_ than SG, while no significant differences were observed for AUC_last_. Both procedures induced profound and persistent alterations in ethanol pharmacokinetics over the three-year period: C_max_ and AUC_last_ approximately doubled, and T_max_ was reduced to about half the preoperative value. C_max_ occurred within 9–15 min compared to 25–29 min before surgery. Patients also had concomitant reductions in total body water, averaging 3.2 kg after SG and 4.8 kg after RYGB at 12 months.

**Conclusion:**

Both RYGB and SG permanently altered ethanol pharmacokinetics, resulting in faster absorption, higher peak concentrations, and higher systemic exposure of alcohol. These changes, which were more pronounced after RYGB, may increase the risk of alcohol use disorders post-surgery. Awareness of the pharmacokinetic effects on RYGB and SG may be relevant for patient education, surgical decision-making, and postoperative monitoring.

**Clinical trial registration:**

This study was registered in ClinicalTrials.gov on May 15 2013. Identifier: NCT01840020.

## Introduction

Despite strong evidence supporting the efficacy of bariatric surgery both for weight loss and metabolic disorders [1], substantial research points to alcohol use disorder (AUD) as a potential complication. Analyses of longitudinal observational data suggest an elevated risk of AUD following the Roux-en-Y gastric bypass (RYGB) procedure [2, 3]. Furthermore, the incidence of AUD after RYGB seems higher compared to other bariatric procedures such as gastroplasty and gastric banding [3–5]. Also, when compared to sleeve gastrectomy (SG), RYGB has less favorable outcomes. In a registry-based study of 17 800 Norwegians who underwent RYGB or SG between 2008 and 2018, we found that patients who received RYGB had 69% higher risk of alcohol-related diagnoses compared to patients with SG [6]. However, because these comparisons rely on observational cohorts with non-random allocation to surgical procedure, selection bias cannot be excluded, and the true risk after the procedures are not known.

Potential changes in the pharmacokinetics of ethanol induced by surgery may contribute to increased risk of AUD. Studies have shown that the maximum concentration of ethanol (C_max_) increased after RYGB [7–9], while the time to reach maximum concentration (T_max_) were shortened [8]. Less conclusive findings were reported in patients after sleeve gastrectomy (SG): Two studies showed increased C_max_ [10, 11], while two studies found no differences [12, 13] after SG. These inconsistencies may reflect methodological limitations in the SG literature. As Acevedo et al. [10] showed, breath alcohol-based estimation may fail to capture C_max_ in bariatric patients because peaks occur within minutes of ingestion. Accordingly, studies need frequent blood sampling in the absorption phase to characterize C_max_ and T_max_ accurately.

The elimination of moderate to high amounts of ethanol follows zero-order kinetics [14]. While the liver plays an essential role in metabolizing ethanol, the gastric mucosa also significantly contributes to the pre-systemic part of the first-pass metabolism [15] due to the expression of alcohol dehydrogenase (ADH) isoenzyme ADH7 [16]. The extent of gastric oxidation of ethanol depends on several factors; it is less prominent in women [17] and individuals with a history of alcohol misuse [17, 18]. Furthermore, first-pass metabolism capacity appears dose-dependent, being most pronounced at alcohol consumption levels associated with low to moderate intake, as seen in typical social drinking contexts [18].

Given conflicting research findings on SG’s impact on ethanol pharmacokinetics, and since SG accounts for a large proportion of bariatric surgeries [19], it is necessary to clarify to what extent SG affects ethanol pharmacokinetics relative to RYGB. This study aimed to compare ethanol pharmacokinetics before and after the two procedures over a period of 3 years.

## Methods

### Study design

BAR-TRIAL was a prospective, longitudinal, non-randomized controlled study assessing oral ethanol bioavailability in patients undergoing RYGB and SG, measured preoperatively and at 3, 12, and 36 months postoperatively.

### Outcomes

The primary outcome was whether the procedures were bioequivalent at 12 months postoperativly. Secondary outcomes were comparison of preoperative and postoperative C_max_, T_max_, AUC_last_, and potential sex differences.

### Participants

The participants were recruited from St. Olavs Hospital, Trondheim University Hospital and Namsos Hospital in Central-Norway. All patients underwent a mandatory group education, including a 45-min session on post-surgical AUD-risks, possible mechanisms and alcohol consumption precautions.

Eligible patients were adults accepted for bariatric surgery, with a BMI > 40 kg/m^2^ or BMI > 35 kg/m^2^ with obesity related comorbidities. Exclusion criteria included a history of alcohol use disorder; abstainers; pregnant or breastfeeding women; and liver disease other than metabolic dysfunction associated steatotic liver disease. Other exclusion criteria were the use of medications such as H2-antagonists, aspirin, metoclopramide, anticholinergic drugs, or any drug which may interact with ethanol and depress the central nervous system. To identify undetected drinking problems, participants completed the Alcohol Use Disorder Identification Test (AUDIT). Scores of 8 or higher indicated hazardous or harmful alcohol use [20] and disqualified for participation. Participants who reported the onset of alcohol problems during the study period were excluded from further testing and were offered a consultation with a study-affiliated psychiatrist and/or referral to an addiction rehabilitation clinic.

Bioequivalence studies typically require a minimum of 12 subjects per group [21]. However, because we had to conduct baseline tests weeks before the surgeon decided on surgical method, we recruited 33 participants to ensure an adequate number in each group. The study coincided with SG supplanting RYGB as the dominant procedure at the recruiting hospitals.

Participants were operated with either Roux-en-Y gastric bypass or sleeve gastrectomy. The RYGB is a surgical procedure in which the stomach is reduced to a small pouch, and this is connected directly to the jejunum, so the stomach and duodenum is bypassed. Where as in sleeve gastrectomy the stomach is reduced to about 25% of its original size, by surgical removal of the greater curvature. There were no randomization to the surgical procedure, but the treating surgeon chose which procedure to be most suitable for each patient based on local guidelines at the time of the study.

### Study procedures

#### Preparations

Participants fasted for at least 4 h before testing, but could drink water ad libitum to maintain hydration. Tests were conducted in the Clinical Research Ward at St. Olavs Hospital under the supervision of study nurses. Upon arrival in the morning, the participants were served a standardized breakfast of a crispbread with soft cheese and 150 ml orange juice, amounts resembling the typical postoperative diet. It was an interval of 60 min between the completion of the standardized breakfast and the start of the ethanol challenge test.

Both oral (per os, PO) and intravenous (IV) ethanol challenge tests were performed at each of the four occasions. To eliminate carry-over effects the PO and the accompanying IV test were performed on different days within a week. The test sequence (PO-IV or IV-PO) was randomized at each session using a web-based randomization service (WebCRF, Norwegian University of Science and Technology (NTNU), Trondheim, Norway). Participants received one or two peripheral intravenous catheters for the PO and IV tests, respectively, preferably in high flow veins (v. basilica or v. cephalica). Isopropanol was used for skin disinfection prior to catheterization. Weight and body composition (total body water) were measured by bioelectrical impedance analysis (InBody720, Biospace Co., Ltd., Seoul, Korea).

#### Alcohol administration

Ethanol dosages were individually determined based on sex and preoperative total body water to achieve a relatively low blood alcohol concentration. The calculated absolute dose for each participant was then repeated at all postoperative assessments (3, 12, and 36 months), i.e., doses were not recalculated based on postoperative reductions in total body water. This precaution ensured consistency across time points. Dosages were calculated as total body water (kg) multiplied by 0.4 g/kg or 0.5 g/kg ethanol for women and men, respectively.

For oral administration, a 1:1 mix of 40% vodka and orange juice was used. Participants consumed the cocktail in small, evenly paced mouthfuls over five minutes, supervised and timed by a study nurse. Alcohol intake occurred one hour after the standardized breakfast. For intravenous administration, a 70% ethanol solution was diluted with 5% glucose to achieve a concentration of 5 g/dL and infused over 30 min via a pump.

#### Blood sampling

Upon arrival and before the challenge test, a blood sample was collected to confirm the absence of alcohol in the patient’s blood. Heparinized blood samples for ethanol analysis were collected from the peripheral intravenous catheter. Preoperative blood samples were collected at the following time points (minutes): 0, 4, 8, 12, 16, 20, 24, 28, 32, 36, 40, 50, 60, 75, 90, 120, 150, 180, 210, 240. To account for an expected faster T_max_ after surgery and to prevent C_max_ from coinciding with the first sample, three additional time points (2, 6, and 10 min) were added in postoperative testing. Time zero (0) corresponds to the moment the patient began drinking or the infusion was initiated. Actual time point, and not scheduled times were used for the analytical procedures.

#### Biochemical analyses

Blood ethanol concentrations were analyzed by a validated method at the university hospital’s Department of Medical Biochemistry. Analyses were conducted using Roche Modular P (Roche Diagnostics, USA) before June 22, 2016, and Adiva Chemistry XPT (Siemens, Germany) thereafter. The detection limit for ethanol was 2.2 mmol/l.

#### Statistical analyses

Independent *t* tests were performed to compare demographic and anthropometric data between the surgical interventions. Paired *t* tests were used to assess changes in body mass before surgery and at 12 months postoperatively. Data was described as mean with either standard deviation (SD) or 95% confidence intervals (95%CI) if not specified otherwise.

Pharmacokinetic parameters (maximum plasma concentration (C_max_)), time to reach maximum plasma concentration (T_max_) and area under the time-concentration-curve from time of administration to last detectable measurement (AUC_last_) by the linear trapezoidal method, were analysed by non-compartmental techniques with Win-Nonlin Standard 8.0 (Pharsight Corporation, USA). Bioequivalence was calculated for the procedures at 12 months with SG as reference. AUC_last_, C_max_ and T_max_ were log-transformed and computed by a two-sided *t* test assuming equal variances. Two drugs are considered bioequivalent if the 90% confidence intervals of the ratios of their pharmacokinetic parameters fall within the range of 80–125% [21].

A mixed model analysis was conducted to compare ethanol pharmacokinetics at different time points while adjusting for sex, age, total body water, muscle mass and the interaction between time point and surgical procedure. The statistical analyses were conducted using SPSS Statistics (Version 29.0, IBM Corp., Armonk, NY, USA).

#### Safety

Blood glucose levels were measured prior to testing and one hour after alcohol intake to monitor for hypoglycemia. Women of childbearing potential underwent a urine pregnancy test prior to each alcohol administration session to rule out pregnancy. Participants stayed in ward until ethanol was no longer detectable in blood. Participants also consented to refrain from driving motorized vehicles on the days of testing. An additional alcohol history was taken prior to scheduling testing dates.

Based on a risk-proportionality approach, 20% of all source data were verified by an independent, certified monitor from the St. Olavs Hospital’s Research Department.

## Results

**Fig. 1:**
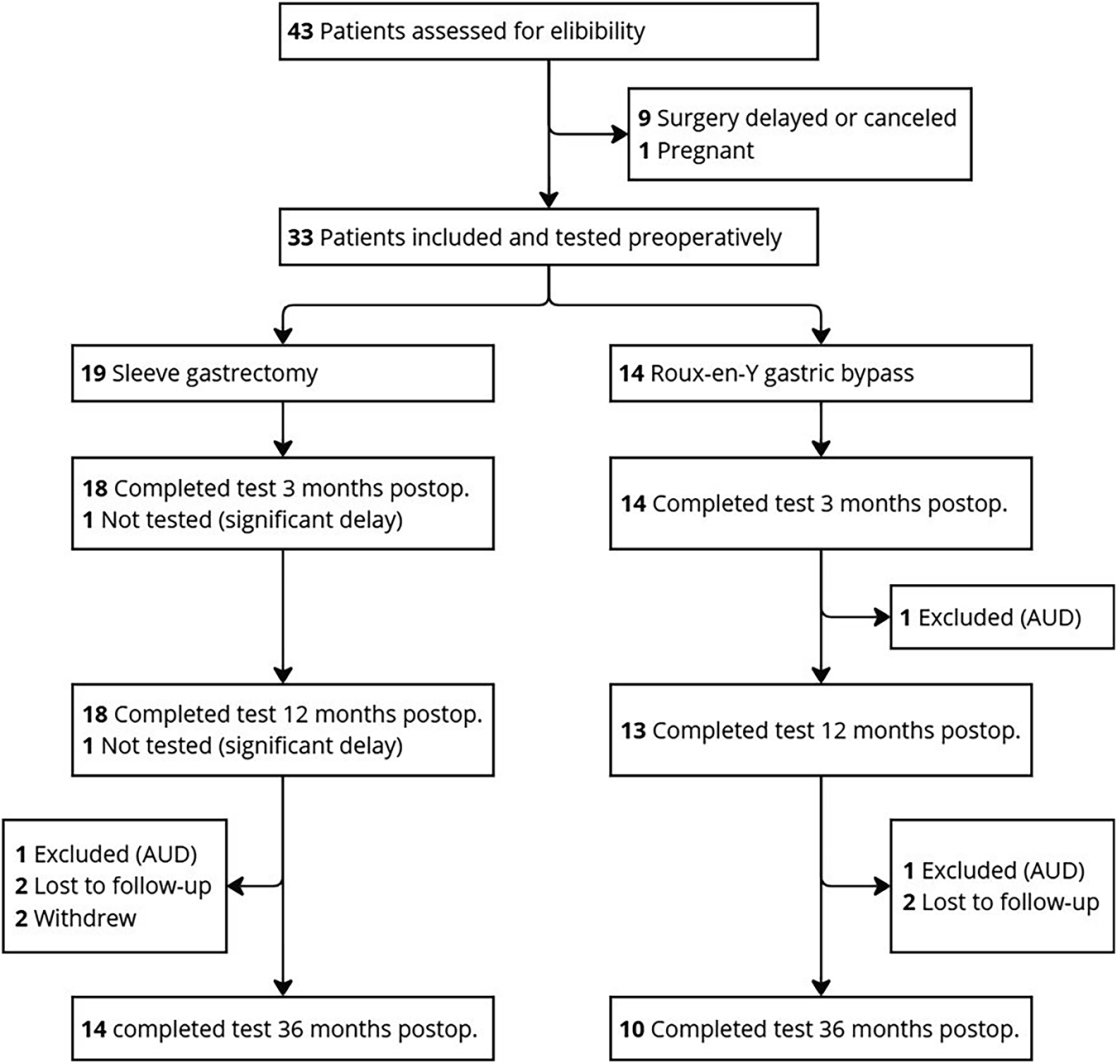
Flow chart of the participants of the trial. *AUD* alcohol use disorder.

Thirty-three patients (19 SG and 14 RYGB, 76% women) participated in this study conducted between 2013 and 2020. Figure 1 shows the enrollment and completion. Completion rates at follow up at 3 months, 1 year, and 3 years were 97%, 94% and 73%, respectively. During the study, two participants (6%) withdrew after 1 year. Three participants (9%) were excluded due to developing AUD: One RYGB patient shortly after the 3-month follow-up and two patients (one RYGB and one SG) after the 1-year follow-up. An additional four participants (12%) were lost to follow-up after 1 year; their AUD status is unknown. No adverse events occurred during testing.

On average, the patients lost 36.2 kg (SD = 11.4) from baseline to 12 months postoperatively (*p* < 0.001), corresponding to a 12.4 decrease in BMI. Table 1 shows a comparison of demographic and anthropometric data between the two surgical groups over the 36 months period. RYGB patients were 7.1 years younger (*p* = 0.042) and had 10.8 kg less body fat three years after surgery (*p* = 0.041) than SG patients. All other between groups-comparisons were statistically non-significant. Both surgical procedures led to significant reductions in total body water at the 12 months postoperative test. RYGB-patients lost an average of 4.8 kg total body water (SD 2.4, t(11) = 7.0, *p* < 0.001), while SG-patients 3.2 kg (SD 1.7, t(17) = 8.0, *p* < 0.001) from baseline to 12 months postoperatively.

**Table 1:** Patient characteristics (*n* = 33).

	Roux-en-Y gastric bypass	Sleeve gastrectomy
Preoperatively	*n* = 14	*n* = 19
Women, % (n)	78.6 (11)	73.7 (14)
Age, years (SD)	36.7 (10.6)	43.8 (8.5)*
Body weight (SD)	118.0 (18.4)	118.7 (17.3)
BMI (kg/m^2^) (SD)	41.0 (4.1)	40.6 (3.3)
Total body water (l) (SD)	45.3 (10.4)	45.7 (7.5)
Body fat (kg) (SD)	56.5 (10.2)	56.5 (8.7)
3 months follow-up	*n* = 12	*n* = 16
Body weight (SD)	90.4 (14.5)	96.5 (14.1)
BMI (kg/m^2^) (SD)	32.3 (4.3)	33.1 (3.0)
Total body water (l) (SD)	39.3 (6.6)	42.0 (6.4)
Body fat (kg) (SD)	36.9 (11.0)	39.2 (8.5)
12 months follow-up	*n* = 12	*n* = 18
Body weight (SD)	79.2 (14.7)	87.3 (16.2)
BMI (kg/m^2^) (SD)	26.9 (3.7)	29.6 (3.9)
Total body water (l) (SD)	42.2 (9.3)	43.0 (6.6)
Body fat (kg) (SD)	21.7 (9.2)	28.7 (9.7)
36 months follow-up	*n* = 10	*n* = 14
Body weight (SD)	87.1 (18.5)	93.3 (18.7)
BMI (kg/m^2^) (SD)	28.2 (4.8)	32.0 (5.3)
Total body water (l) (SD)	44.2 (9.4)	41.4 (7.5)
Body fat (kg) (SD)	26.1 (11.3)	36.9 (12.7)*

Differences were analysed with independent *t* tests (* *p* < 0.05).

Note: Alcohol doses were calculated from each participant’s preoperative total body water and the same absolute dose was repeated at all postoperative assessments.

*BMI* body mass index.

The mean ethanol test dose, calculated from preoperative total body water, was 19.6 g (28.8 g for men and 16.7 g for women). When stratified by surgical procedure, the average dose was 19.4 g in the RYGB group and 19.7 g in the SG group.

Bioequivalence was calculated from the preoperative test to the 12-month postoperative test with SG serving as the reference (Table 2). The geometric mean ratios (90%CI) were for AUC_last_ 0.97 (0.80–1.19); C_max_ 1.27 (1.06–1.55); and T_max_ 0.69 (0.54–0.89).

**Table 2:** Primary pharmacokinetic variables of ethanol administered orally in patients undergoing Roux-en-Y gastric bypass (*n* = 14) or sleeve gastrectomy (*n* = 19).

	Preoperatively		3 months postoperatively		12 months postoperatively		36 months postoperatively	
	RYGB	SG	RYGB	SG	RYGB	SG	RYGB	SG
C_max_ (mmol/l)	8.5 (6.8, 10.3)	8.4 (6.7, 10.1)	18.6 (15.9, 21.3)	17.4 (15.0, 19.7)	21.0 (18.1, 23.9)	16.2 (13.7, 18.7)	17.1 (13.5, 20.8)	15.6 (12.4, 18.8)
T_max_ (minutes)	25.8 (21.1, 30.5)	29.0 (24.9, 33.2)	11.7 (8.5, 14.8)	12.1 (9.4, 14.8)	9.6 (6.4, 12.8)	14.2 (11.5, 17.0)	9.8 (6.4, 13.2)	15.8 (12.7, 18.9)
AUC_last_	493 (399, 587)	418 (329, 507)	1020 (891, 1149)	976 (863, 1089)	971 (848, 1094)	938 (833, 1042)	845 (713, 976)	834 (718, 950)

Data are given as mean (95%CI).

*RYGB* Roux-en-Y gastric bypass, *SG* sleeve gastrectomy, *C*_*max*_ maximum plasma concentration, *T*_*max*_ time to maximum plasma concentration, *AUC*_*last*_ area under the concentration-time curve from time of alcohol administration to last measurable concentration.

**Fig. 2:**
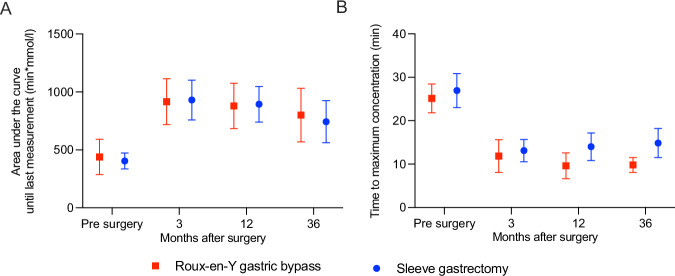
Changes in ethanol exposure and time to maximum concentration after bariatric surgery. **A** Change in the total exposure (AUC_last_) to oral ethanol intake before and after Roux-en-Y gastric bypass or sleeve gastrectomy. **B** Change in time to maximum concentration (T_max_) to oral ethanol intake before and after Roux-en-Y gastric bypass and sleeve gastrectomy.

The total exposure to ethanol (AUC_last_) for the oral test increased significantly after both surgical procedures and remained elevated throughout the 3-year period (Fig. 2A). Time-concentration curves for intravenous ethanol did not differ before and after surgery (Supplementary File 1). On the other hand, the time to maximum concentration (T_max_) after oral intake were much faster postoperatively, being 9–15 min compared to 25–29 min before operation (Table 2/Fig. 2B).

**Fig. 3:**
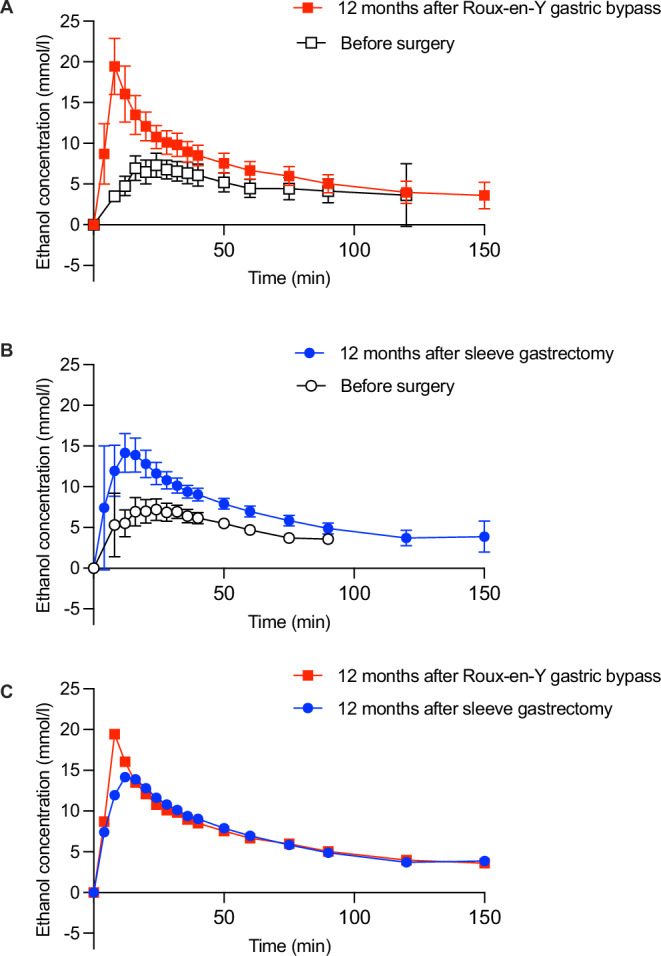
Ethanol concentration–time profiles before and after Roux-en-Y gastric bypass and sleeve gastrectomy. **A** Time course of blood concentrations of ethanol before surgery (white squares) and 12 months after surgery with Roux-en-Y gastric bypass (red squares). **B** Time course of blood concentrations of ethanol before surgery (white dots) and 12 months after surgery with sleeve gastrectomy (blue dots). Presented as mean with 95% confidence intervals in (**A**, **B**). **C** Time course of blood concentrations of ethanol 12 months after surgery with Roux-en-Y gastric bypass (red squares) or sleeve gastrectomy (blue dots). Confidence intervals are left out for clarity.

The C_max_ increased following both surgical procedures, accompanied by a much faster T_max_ than before surgery (Fig. 3A, B, Table 2). These effects were maintained at the 3-year follow-up (Table 2, Supplementary File 2).

Participants who underwent RYGB exhibited higher C_max_ and shorter T_max_ 12 months after surgery compared to participants who underwent SG (Fig. 3C).

The mixed model analyses of the complete data material from the oral tests showed no effect of surgical method for AUC_last_ [F(1,98.998) = 1.010; *p* = 0.317]. However, surgical method came up as a significant main effect for T_max_ [F(1,103.383) = 8.476; *p* = 0.004] together with time passed after surgery [F(3,54.653) = 29.658; *p* < 0.001] when controlling for sex, age, total body water, and muscle mass. Sex did not contribute significantly to T_max_ [(F(1,98.743) = 0.346); *p* = 0.558]. This means T_max_ was consistently shorter following RYGB compared to SG and also markedly reduced at all postoperative time points relative to preoperative measurements. Surgical method [F(1,87.623) = 4.167; *p* = 0.044] and timepoint [F(3,46.469) = 48.289; *p* < 0.001] came up as significant main effects also for C_max_; sex was not significant for C_max_ [(F(1,61.853)) = 2.138; *p* = 0.149]. Hence, C_max_ was higher after RYGB compared to SG, and stayed elevated at all postoperative time points compared to preoperative levels. None of the mixed model analyses found any significant interaction effects.

## Discussion

This is the first prospective, longitudinal study comparing the pharmacokinetics of ethanol after two common bariatric procedures; the RYGB and SG. Our findings show that both procedures approximately doubled both C_max_ and the oral bioavailability of ethanol, while T_max_ was reduced to about half the preoperative value. The change in pharmacokinetics was even more pronounced for RYGB compared to SG with both shorter time to maximum concentration and much higher concentrations over all. These changes appear to be permanent.

Regulatory bodies apply bioequivalence calculations of AUC_last_, C_max_, and T_max_ to determine whether a generic drug can be considered equal to the original drug requiring that the 90% confidence interval of the test-to-reference ratio should fall within 80–125% [21]. This principle was applied to determine if there was a clinically relevant difference between the two surgical procedures. AUC_last_ was bioequivalent and similar for the procedures, while this was not true for C_max_ of (27% higher) and T_max_ (31% shorter) after RYGB compared to SG. This was confirmed by the mixed model analysis which was adjusted for common factors that might have contributed the changes observed.

Both RYGB and SG accelerate gastric emptying of liquids [22, 23], which likely contributes significantly to the reduced T_max_, the increased AUC and the more rapid C_max_. The relative differences in systemic ethanol exposure between the procedures may partly be attributable to both pharmacokinetic and distinct mechanistic effects. Postoperatively, total body water decreased by about 10%, slightly raising the effective ethanol dose per liter of body water. However, doses were calculated from preoperative total body water and kept constant across follow-ups, and the total body water reduction was moderate and similar between RYGB (–3.1 L) and SG (–2.7 L) at 12 months. The intravenous tests further showed unchanged elimination, indicating that the observed increases in C_max_ and decreases in T_max_ mainly reflect altered gastric first-pass metabolism, absorption, and distribution rather than dosing or elimination.

In the present experiment, ethanol was administered in the “fed” state, i.e. 60 min after a standardized breakfast. This context may be relevant when comparing RYGB and SG. Unlike RYGB, SG is a pylorus-preserving procedure, and consumption of food before drinking is likely to delay gastric emptying to a greater extent after SG than after RYGB. This may contribute to the more pronounced effects on C_max_ and T_max_ observed after RYGB in our study. It is conceivable that when alcohol is consumed in the fasted state, the difference in ethanol pharmacokinetics between the two procedures would be smaller.

RYGB results in less residual gastric mucosa, as the stomach volume is reduced to only 15-30 ml compared to 150–200 ml after SG. We hypothesize that patients undergoing SG may retain some residual gastric first-pass metabolism compared to patients undergoing RYGB. The emptying of liquids into the jejunum occurs almost immediately following RYGB [24], which also contributes to the more rapid uptake after this procedure. Although parts of the jejunum are bypassed in RYGB, it is unlikely that this reduces intestinal ethanol absorption, as the absorptive capacity of the remaing intestine is obviously sufficient.

Seventy-six percent of the participants were women, reflecting the typical composition of bariatric patients. Gastric first-pass metabolism of ethanol is considered sex-dependent, with women exhibiting less gastric ADH activity than men, leading to higher oral bioavailability [17]. However, our regression analyses of T_max_ and C_max_ found no significant sex difference. One possible explanation is that both women and men possess significant gastric first-pass metabolism, but our study lacked the power to detect a difference. Given reports that women also have gastric first-pass metabolism [25], the observed changes could be due to the elimination of this protective metabolic mechanism.

### Increasing the reinforcing effects of alcohol

Both RYGB and SG increased C_max_ while reducing T_max_. Even a change in only one of these parameters may intensify the effects of alcohol. Given the documented elevated risks for AUD after bariatric surgery [2, 3, 5]—which likely underestimate the true prevalence—the altered pharmacokinetics may be a central mechanism underlying postoperative AUD development.

A key factor in psychoactive drugs’ addictive potential is the rapidity with which they enter the bloodstream, and consequently the brain, producing a “high”. For instance, smoking cocaine has a higher addiction risk compared to snorting [26]. In bariatric surgery patients, the pharmacokinetic changes are analogous to switching to a faster administration route for alcohol. However, in the case of cocaine, the transition from snorting to smoking is a deliberate choice made by the user to achieve a stronger intoxicating effect. Bariatric patients drinking alcohol, on the other side, do not change their method of use; the intensified effects is simply a result of the surgery they have undergone. Given that alcohol is a legal and easily accessible substance, bariatric surgery patients may unknowingly engage in hazardous drinking, even at levels commonly perceived as low-risk. The heightened and repeated peaks in blood alcohol concentration (“highs”) they may experience even during social drinking, could place them at significant risk. In contrast, their consumption may appear moderate to others.

Although AUC_last_ was bioequivalent between RYGB and SG, acute risk of alcohol-related harm is driven more by how high the alcohol peak is (C_max_) and how fast it is reached (shorter T_max_) than by total exposure. The Mellanby effect—i.e., acute tolerance within the same drinking episode—means that at the same BAC, people typically feel more intoxicated on the rising limb than on the falling limb [27]. In line with this, patients after bariatric surgery frequently report rapid onset and offset of intoxication, sometimes with multiple peaks during an evening [28], and epidemiological data show increased accident-related mortality after RYGB [29]. Together, these observations imply two practical consequences: (i) a rapid rise produces stronger subjective effects and greater risk-taking, and (ii) on the descending limb people may feel «sober» sooner than they actually are, leading to misjudgments (e.g., deciding to drive) while objective performance remains impaired. Given that RYGB produced a higher C_max_ and shorter T_max_ than SG, these kinetics plausibly translate into stronger acute pharmacodynamic effects despite similar AUC, consistent with our registry finding of ~70% higher rates of alcohol-related diagnoses after RYGB versus SG [6].

Because all BACs were obtained from peripheral venous samples, our postoperative measurements during the rapid absorption phase likely underestimate arterial—and thus cerebral—exposure. Arterio-venous gradients are minimal when absorption is slow, but the markedly shortened T_max_ after metabolic surgery renders this difference clinically relevant, implying that the true peak exposure at the brain is higher than suggested by our estimates of venous C_max_ [30].

### Clinical implications

The fact that at least 3 participants (9%) developed AUD during the study—despite receiving patient education and information about the rationale of the study—underscores the criticial nature of this issue. Notably, none of the three had their alcohol issues detected by the operating clinics’ follow-up program, emphasizing the necessity of both enhanced awareness and systematic screening and follow-up in clinics performing bariatric surgery.

Understanding how SG and RYGB alter alcohol pharmacokinetics is crucial for informed medical decisions and patient education before bariatric surgery. Both procedures lead to faster, higher, and longer-lasting effect of alcohol intake provided that alcohol consumption patterns remain similar to before surgery. This suggest that patients should be advised to significantly reduce their alcohol intake after surgery and to extend the time taken per unit of alcohol consumption.

Insight into the distinct pharmacokinetic profiles of SG and RYGB enables personalized treatment. However, any comparison should be framed by the shared risk: both procedures reliably produce faster and higher alcohol exposure, consistent with increased vulnerability to hazardous drinking and AUD. Accordingly, in patients with multiple substance-misuse risk factors, clinicians should critically reassess the indication for bariatric surgery and, in some cases, consider not operating. Between procedures, the steeper rise and higher peak after RYGB plausibly confer greater acute risk than SG; nevertheless, other clinical considerations may still justify RYGB in selected cases. Regardless of procedure, patients should be explicitly counseled about these risks and systematically monitored postoperatively for AUD. For patients at known high risk, non-surgical options—including pharmacological weight-loss treatment—should be prioritized.

In a postoperative context, patients may find their prior strategies for drinking responsibly ineffective due to these physiological changes. They may prefer to avoid beer due to carbonation discomfort while struggling to control intoxication levels with strong drinks due to rapid absorption. Additionally, reduced capacity to eat and drink simultaneously makes it challenging to avoid drinking on an empty stomach—a precaution that can mitigate rapid intoxication. Susceptibility to blackouts further complicates responsible drinking after bariatric surgery [31].

For clinicians, the increased systemic exposure of ethanol has practical consequences for alcohol history-taking during patient follow-up. Our findings may have implications for the use of screening instruments that assess alcohol consumption. A commonly used tool, the AUDIT (Alcohol Use Disorders Identification Test) [20], starts with questions like “How many drinks containing alcohol do you have on a typical day when you are drinking?” and “How often do you have six or more drinks on one occasion?”. As the scoring relies on assumptions of the systemic exposure following normal physiology, the total score may underestimate the health risk associated with actual alcohol exposure in bariatric surgery patients. In other words, the use of AUDIT after bariatric surgery may result in false negatives.

### Strengths and limitations

Our study uniquely compared SG and RYGB under similar circumstances with long-term, prospective data, accounting for inter-individual variations in alcohol metabolism–unlike previous cross-sectional studies [7, 8, 10]. Using frequent blood sampling instead of breathalyzers [12, 13] allowed for more precise C_max_ and T_max_ estimation. Incorporating body composition measurements allowed for controlling for the effect of altered body water content. A limitation was that we did not obtain arterial or arterialized venous samples which may have made our samples taken early post-ingestion to underestimate the cerebral exposure to ethanol. All ethanol challenge tests were conducted in the fed state, 60 min after a standardized meal, which reflects typical real-life drinking situations where alcohol is consumed with food. This may, however, limit generalizability to fasted alcohol intake and could accentuate differences between RYGB and the pylorus-preserving SG. The lack of randomization between RYGB and SG is yet another limitation.

## Conclusions

Both RYGB and SG significantly and permanently alter pharmacokinetics of ethanol by significantly increasing the total systemic exposure and maximum plasma concentration of ethanol while simultaneously reducing the time to reach it. The effects were more pronounced after RYGB than SG. These changes may contribute to the elevated risk of postoperative alcohol use disorders. These facts should be included in preoperative patient education, informed surgical decision-making, and postoperative monitoring for AUD.

## Supplementary information

Supplementary file 1

Supplementary file 2

## Data Availability

The data supporting the findings of this study can be made available upon reasonable request from the corresponding author.
