# Supplementary material for: Ethanol pharmacokinetics before and after sleeve gastrectomy and Roux-en-Y gastric bypass: a 3 year prospective study (the BAR-TRIAL)
Source: Int J Obes (Lond). 2026 Jun 17;50(7):1573–80. doi: 10.1038/s41366-026-02113-3 (PMC13391354; doi:10.1038/s41366-026-02113-3)

Supplementary file 1

Intravenous ethanol before and after Roux-en-Y gastric bypass and sleeve gastrectomy

A

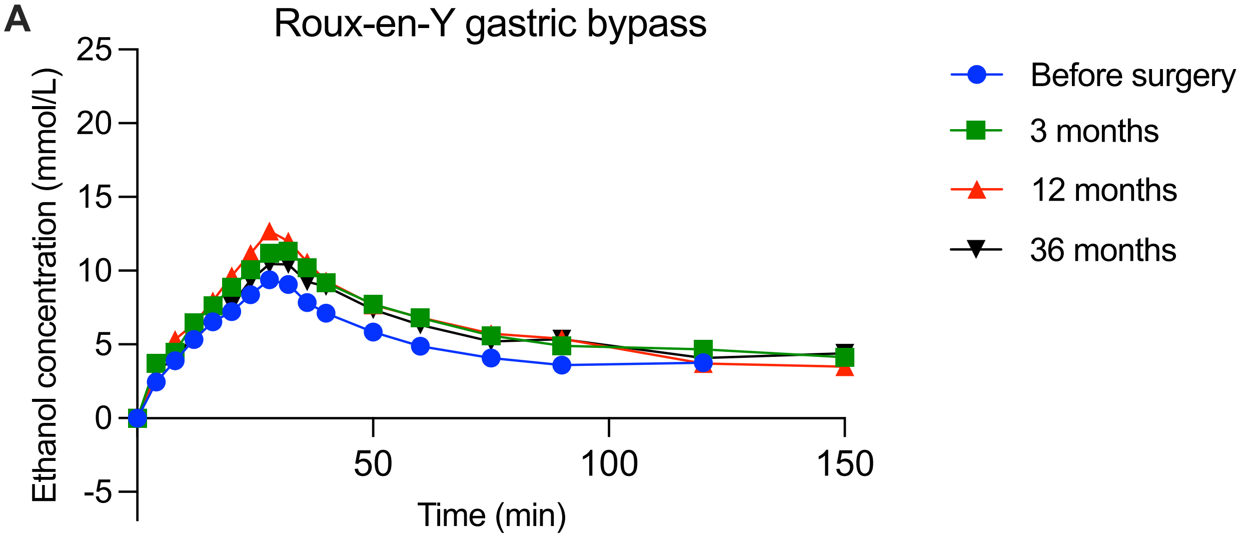

B

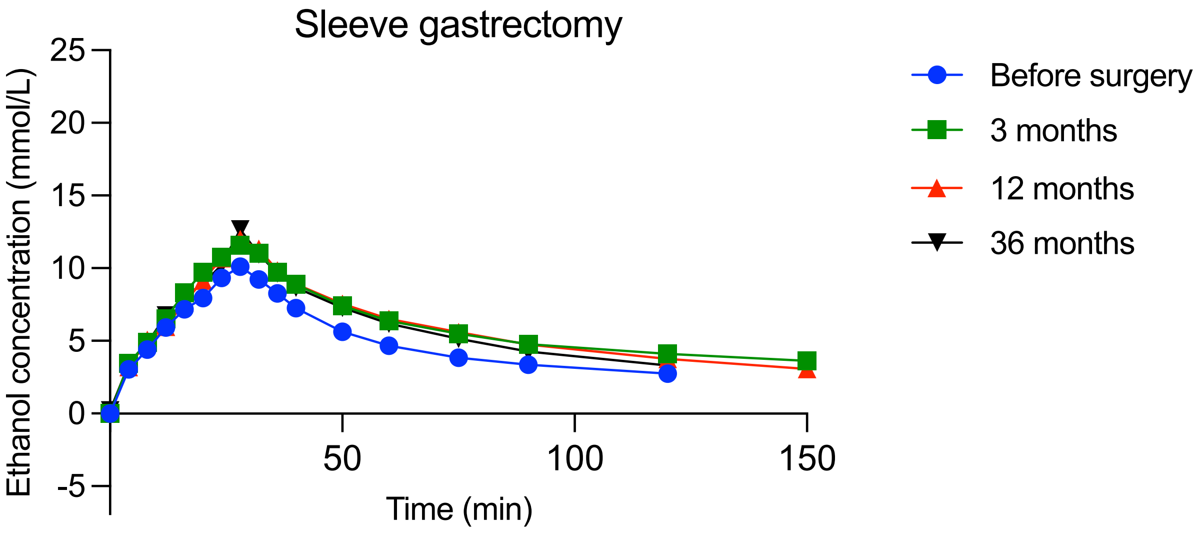

Supplement: Supplementary file 1 [file 41366_2026_2113_MOESM1_ESM.pdf]
